# Prevalence and clinical impact of frailty in COPD: a systematic review and meta-analysis

**DOI:** 10.1186/s12890-023-02454-z

**Published:** 2023-05-12

**Authors:** Lina Wang, Xiaolin Zhang, Xinmin Liu

**Affiliations:** Geriatric Department, Peking University First Hospital, Xicheng District, Xishiku Avenue No 8, Beijing, 100034 China

**Keywords:** Chronic obstructive pulmonary disease, Frailty, Mortality, Prevalence, meta-analysis

## Abstract

**Background:**

Frailty has been increasingly identified as a risk factor of adverse outcomes in chronic obstructive pulmonary disease (COPD). The prevalence and impact of frailty on health outcomes in people with COPD require clarification.

**Methods:**

PubMed, Embase, The Cochrane Library and Web of Science (January 1, 2002, to July 1, 2022) were comprehensively searched to identify studies related to frailty and COPD. Comparisons were made between people who did and did not have frailty for pulmonary function, dyspnea severity, 6-minute walking distance, activities of daily life, and mortality.

**Results:**

Twenty studies (9 cross-sectional, 10 cohort studies,1 clinical trial) from Europe (9), Asia (6), and North and South America (4), Oceania (1) involving 11, 620 participants were included. The prevalence of frailty was 32.07% (95% confidence interval (CI) 26.64–37.49) with a range of 6.43–71.70% based on the frailty tool used. People with frailty had lower predicted forced expiratory volume in the first second (mean difference − 5.06%; 95%CI -6.70 to -3.42%), shorter 6-minute walking distance (mean difference − 90.23 m; 95%CI -124.70 to -55.76), poorer activities of daily life (standardized mean difference − 0.99; 95%CI -1.35 to -0.62), higher CAT(COPD Assessment Test) score(mean difference 6.2; 95%CI 4.43 to 7.96) and mMRC (modified Medical Research Council) grade (mean difference 0.93; 95%CI 0.85 to 1.02) compared with those who did not (*P* < 0.001 for all). Meta-analysis showed that frailty was associated with an increased risk of long-term all-cause mortality (HR 1.68; 95% CI 1.37–2.05; *I*^2^ = 0%, *P* < 0.001).

**Conclusion:**

Frailty is prevalent in people with COPD and linked with negative clinical outcomes including pulmonary function, dyspnea severity, exercise capacity, quality of life and mortality.

**Supplementary Information:**

The online version contains supplementary material available at 10.1186/s12890-023-02454-z.

## Introduction

Frailty is a complex geriatric syndrome characterized by a decline in physiological capacity across several organ systems, accompanied by an increased risk of adverse outcomes including falls, delirium, disability, hospitalization, and mortality in older adults [1]. The Fried frailty phenotype(FFP) [2], clinical frailty scale (CFS) [3], the Hospital Frailty Risk Score (HFRS) [4] and the frailty index are usually used to evaluate the frailty of older persons.

Frailty can predict the negative prognosis of several chronic diseases, such as chronic kidney disease [5], lower extremity peripheral artery disease [6], atrial fibrillation [7] and heart failure [8]. However, the relationship between frailty and chronic obstructive pulmonary disease (COPD) are needed to further clarify. Frailty is common in individuals with COPD. Patients with COPD appear to have an increased risk of presenting frailty. Marengoni et al. [9] found that the pooled prevalence of frailty in individuals with COPD was 19% and patients with COPD had two-fold increased risk of frailty comparing those without COPD. Previous studies indicated that frailty appears to have a negative impact upon clinical outcomes related to function and health [10–13]. Frailty was associated with longer-duration hospitalization, poorer quality of life and higher risk of readmission in patients with COPD [10, 14], but the real clinical impact has not yet been explicitly quantified. Significantly, frailty status in older adults can be improved by the targeted interventions [15]. Further understanding of the relationship between the frailty and COPD may guide the comprehensive management of patients with COPD. In this study, therefore, we aim to conduct a systematic review with meta-analysis to quantify the impacts of frailty upon health outcomes.

## Methods

We performed a systematic review following the Preferred Reporting Items for Systematic Reviews and Meta-Analysis (PRISMA) guidelines 2020 [16]. The protocol for this review was registered in PROSPERO(CRD42022369111).

### Search strategy and inclusion and exclusion criteria

PubMed, Embase, The Cochrane Library and Web of Science were searched for studies using the following free-text and subject heading terms: ‘Pulmonary Disease’, ‘Chronic Obstructive Bronchitis’, ‘chronic obstructive pulmonary disease’, ‘COPD’, ‘Chronic Obstructive Airway Disease’, ‘Chronic Obstructive Lung Disease’, ‘emphysema’, ‘bronchitis’ AND ‘frail elderly’, ‘frail’, ‘frailty’ (Additional Table  1). The most used model to assess the frailty-the phenotype model was developed by Fried et al. in 2001 [2]. Recognition of frailty is becoming increasingly important in recent years. Therefore, the search period was from the January 1, 2002, to July 1, 2022.

The inclusion criteria were: (1) articles in English; (2) the design was a cross-sectional, case-control, prospective, or retrospective cohort study or clinical trial in humans; (3) studies must have been conducted on adults with COPD;(4) patients had a definite diagnosis of frailty, defined according to any criteria provided it was stated in the methodology; (5) studies that provided comparative data between people with COPD who did and did not have frailty, as follows: (a) pulmonary function measured by spirometry (e.g. FEV1% predicted); (b) dyspnea severity including CAT(COPD Assessment Test) and mMRC (modified Medical Research Council) grade; (c) physical function, derived from common clinical assessment including six minutes walking test (6MWT) and activities of daily living(ADL); (d)hospital readmission, acute exacerbation and all-cause mortality.

Articles were excluded if they: (1) did not investigate the aims of the review; (2) were not original (e.g. editorial, review, congress abstract); (3) if frailty was assessed only with a single symptom or measure (e.g. only gait speed or grip strength); and (4) was a duplicate.

### Quality assessment

We used the tool from the Newcastle-Ottawa Scale (NOS) for cohort studies [17] and Agency for Healthcare Research and Quality (AHRQ) scale for cross-sectional studies [18]. For cohort studies, scores > 7 were considered a low risk of bias; 5 to 7, a moderate risk; and < 5, a high risk. Each cross-sectional study was scored as follows: 0–3, low quality; 4–7, medium quality; and 8–11, high quality. The Cochrane Collaboration’s tool for assessing risk of bias was used for randomized controlled trials [19]. Studies with high risk of bias in at least one of the six areas were assumed to have an overall high risk of bias. Two authors (L.W. and X.Z.) independently examined the sources of bias of the included studies and any disagreement was resolved through discussion. A third author (X. L.) was consulted when consensus was not achieved.

### Data extraction and study outcomes

Data from the different studies was extracted in a prespecified spreadsheet in Microsoft Excel. The extracted data elements consisted of (1) name of first author, publication year; (2) design type of study; (3) the sample settings and size; (4) the characteristics of the population, including gender, age, and smoking status; (5) assessment of frailty; (6) number of frailty and non-frailty; (7) the data of hospital readmission, acute exacerbation, and mortality. (8) FEV1% predicted, 6MWT distance, ADL, CAT score, mMRC grade, means (and standard deviation) were extracted.

### Statistical analysis

Where individual studies reported different measurements of frailty, if able to be determined, the most ‘conventional’ type was used. If the studies provide the continuous outcome data as median and interquartile range, we converted the median and interquartile range to mean and standard deviation [20, 21]. Clinical outcome data from studies comparing people with COPD who did and did not have frailty were meta-analyzed via Stata version 17(Stata Corporation, College Station, TX, USA). Continuous outcome data evaluated using homogenous metrics were summarized as mean differences, while data arising from heterogenous metrics were summarized as standardized mean differences (SMDs) and 95% confidence intervals (CI). The impact of frailty on mortality was summarized by pooling the fully adjusted hazard ratio (HR) with 95% CI using a random effect (DerSimonian-Laird) model. Statistical heterogeneity was quantified by using the *I*^2^ statistic (values < 25% considered low, 50–75% moderate, and > 75% high). If moderate or substantial heterogeneity was identified, we used random effects models to pool outcomes. Otherwise, a fixed effects model was used. Publication bias was assessed with the funnel plots and Egger tests. Two-tailed *P* values of < 0.05 were considered statistically significant.

## Results

### Description of included studies

**Fig. 1:**
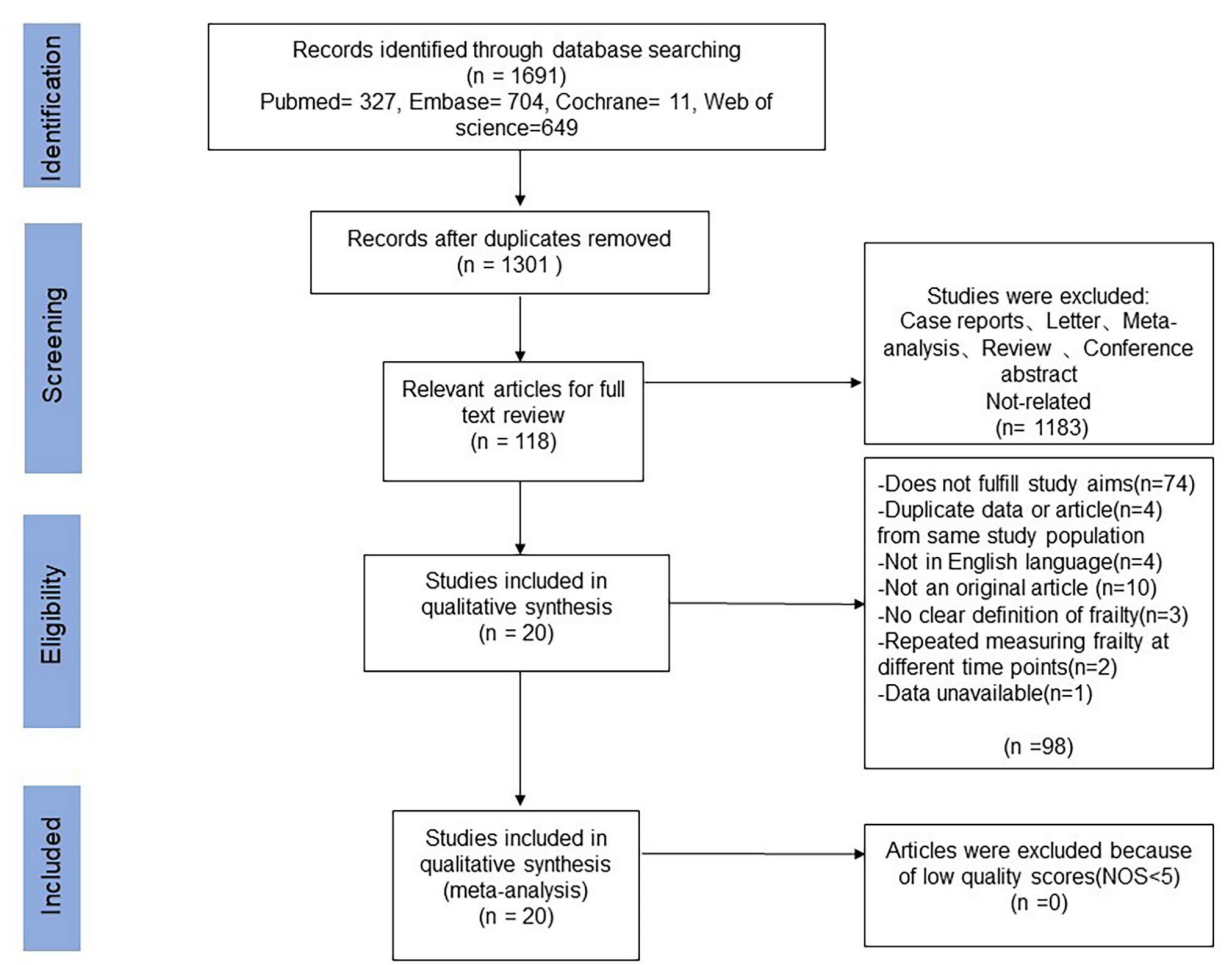
Flow diagram of the study selection process

A flow diagram detailing the literature search is provided in Fig. 1. Of the 1301 abstracts identified during the search, 118 were selected for full-text reading, and 1183 were excluded because they did not relevant to the topic of the review. After reading the full text, twenty articles involving 11, 620 participants were included in the final review. Of these, 10 were observational cohort studies, 9 adopted a cross-sectional design, and one was a randomized clinical trial. Characteristics of included studies are presented in Table 1. These studies were conducted during a diverse range of populations, including nine studies from Europe, six from Asia, four from North and South America and one from Oceania.

**Table 1:** Characteristics of the included studies regarding the frailty and clinical outcomes of COPD

First author/Year	Country	Design	Settings	Sample size	Frailty measure	Age(year)	Male(N, %)	Smoking status(never/former/current, N)	GOLD (I/II/III/IV, N)	Frailty prevalence, (N, %)
Finamore 2021 [22]	Italy	Clinical trial	Patients with stable COPD (GOLD I-III)	53	PRISMA-7: score equal or above 3 indicates individuals with an increased risk to be frail; TUG: A cutoff of 10 s has demonstrated to be sensitive in discriminating frail individuals	73 ± 8	26,49%	NA	1/35/17/0	38, 71.70%
Kagiali 2022 [23]	Turkey	Cross-sectional	Patients with stable COPD	48	FFP	Frail group: 67.35 ± 5.13; Non-frail group: 65.11 ± 4.66	38,79%	NA	NA	20, 41.67%
Kennedy 2019 [10]	America	Cohort-study	Nonsmokers (abstinent > 6 month) with moderate to severe COPD	886	FFP	67 (IQR, 63–70)	563,63.5%	never + former: 886	NA	57, 6.43%
Luo 2021 [11]	China	Cohort-study	Older adults (≥ 65) with stable COPD	309	FFP	86 (IQR: 80–90)	241,78%	143/99/67	114/162/30/3	154, 49.84%
Gale 2018 [26]	UK	Cross-sectional	Community-based patients with stable COPD	520	FI-GCA	Frail: 63.7 ± 8.2; Non-frail: 67.1 ± 7.1	270, 51.9%	NA	NA	143, 27.50%
Galizia 2011 [34]	Italy	Cohort-study	Patients aged 65y with COPD	489	Frailty staging system combining seven core domains of functioning: disability, mobility, cognitive function, visual function, hearing function, urinary continence, and social support.	74.9 ± 6.3	268,54.8%	253/127/109	NA	239, 48.88%
Gephine 2021 [25]	Canada	Cross-sectional	Patients with COPD (aged 40y) with chronic respiratory failure	44	FFP	66 ± 8	30.68%	NA	0/3/18/23	19, 43.18%
Park 2021 [36]	Korea	Cross-sectional	Community-based patients with COPD	417	Six criteria were available including weight loss, physical activity, mobility, hearing, strength for physical frailty, and anxiety/depression for psychological frailty.	65.36 ± 9.35	409,98.1%	0/258/159	207/194/15(1/2/3&4)	148, 35.49%
Kusunose 2017 [27]	Japan	Cross-sectional	Patients with stable COPD	79	The Kihon Checklist	74.8 ± 6.3	NA	Former + current: 79	24/40/12/3	17, 21.52%
Maddocks 2016 [29]	UK	Cohort study	Outpatients with stable COPD	816	FFP	69.8 ± 9.7	484,59.3%	54/624/146	NA	209, 25.61%
Medina-Mirapeix 2018 [32]	Spain	Cross-sectional	Outpatients with stable COPD aged 40–80 years	137	FFP	66.9 ± 8.3	120,87.6%	NA	NA	12, 8.76%
Naval 2021 [24]	Spain	Cross-sectional	Outpatients with stable COPD	127	FFP	66.5 ± 7.9	108,85.0%	NA	1/58/57/9	31, 24.41%
Yee 2020 [13]	USA	Cohort study	Outpatients with COPD	280	FFP	Frail: 68.6 ± 9.2; Prefrail:66.9 ± 8.4; Non-frail:69.4 ± 8.0	224,80%	Current smoker: 76	0/112/115/55	64, 22.86%
Dias 2020 [35]	Brasil	Cross-sectional	Outpatients with COPD	153	FRAIL Scale	Frail: 67.0 (61.0–71.5); Prefrail:70.0 (65.0–73.0); Non-frail:69.5 (60.5–80.5)	84,54.9%	NA	NA	77, 50.33%
Hanlon 2022 [30]	UK	Cohort study	Community-based patients with COPD aged 40–70 years	3011	FFP*; the frailty index	61.9 ± 5.9	1650,54.8%	477/1580/926	1141/993/500/102	514, 17.07%
Hirai 2019 [28]	Japan	Cross-sectional	Older (aged ≥ 65 years) outpatients with COPD	201	The Kihon Checklist*; Japanese version of the Cardiovascular Health Study; Study of Osteoporotic Fractures	76 (IQR: 70–81)	175,87%	NA	46/102/42/10	76, 37.81%
Lahousse 2016 [31]	The Netherlands	Cohort study	Community-based patients with COPD	402	FFP	75 ± 9	229,57%	62/259/81	200/174/28/0	41, 10.20%
Bernabeu-Mora 2017 [14]	Spain	Cohort study	Patients during hospitalization for AECOPD	102	REFS	71.0 ± 9.1	96,93.2%	Active smoker: 35	NA	57, 55.88%
Scarlata 2021 [33]	Italy	Cohort study	Outpatients with stable COPD	150	Frailty index	73 ± 8	107,72%	15/84/51	NA	71, 47.33%
Ushida 2022 [12]	Japan	Cohort study	Patients admitted by ambulance with COPD	3,396	HFRS	75.9 ± 11.2	2703,79.6%	NA	NA	14%

**COPD** Chronic obstructive pulmonary disease, **AECOPD** acute exacerbation of chronic obstructive pulmonary disease, **TUG** Timed “Up and Go” test, **GOLD** Global initiative for chronic Obstructive Lung Disease, **FFP** Fried frailty phenotype, **FI-GCA** the comprehensive geriatric assessment and a frailty index, **REFS** Reported Edmonton Frail Scale, **HFRS** Hospital Frailty Risk Score, **NA** not available. * Select the frailty measurement to analyze

### Quality of included studies

The details of the quality assessment are shown in Additional Tables  2, 3 and 4. The quality assessment showed that of the 20 studies included, one article [22] was of low quality and 6 articles [23–28] were medium quality; the remaining articles were all rated as high quality.

### Methods used to assess frailty

For frailty evaluation, 10 studies [10, 11, 13, 23–25, 29–32](50%) used the criteria of FFP; frailty was also measured by using other measurements, such as the Timed “Up and Go”(TUG) test [22](1, 5%) the comprehensive geriatric assessment (CGA) [26](1, 5%), frailty index [26, 30, 33](3, 15%), Frailty Staging System [34](1,5%), the Kihon Checklist [27, 28](2,10%), FRAIL Scale [35](1,5%), the Reported Edmonton Frailty Scale(REFS) [14](1,5%) and HFRS [12](1,5%). One study used six criteria including weight loss, physical activity, mobility, hearing, strength for physical frailty, and anxiety/depression to assess frailty [36].

### Frailty prevalence

**Fig. 2:**
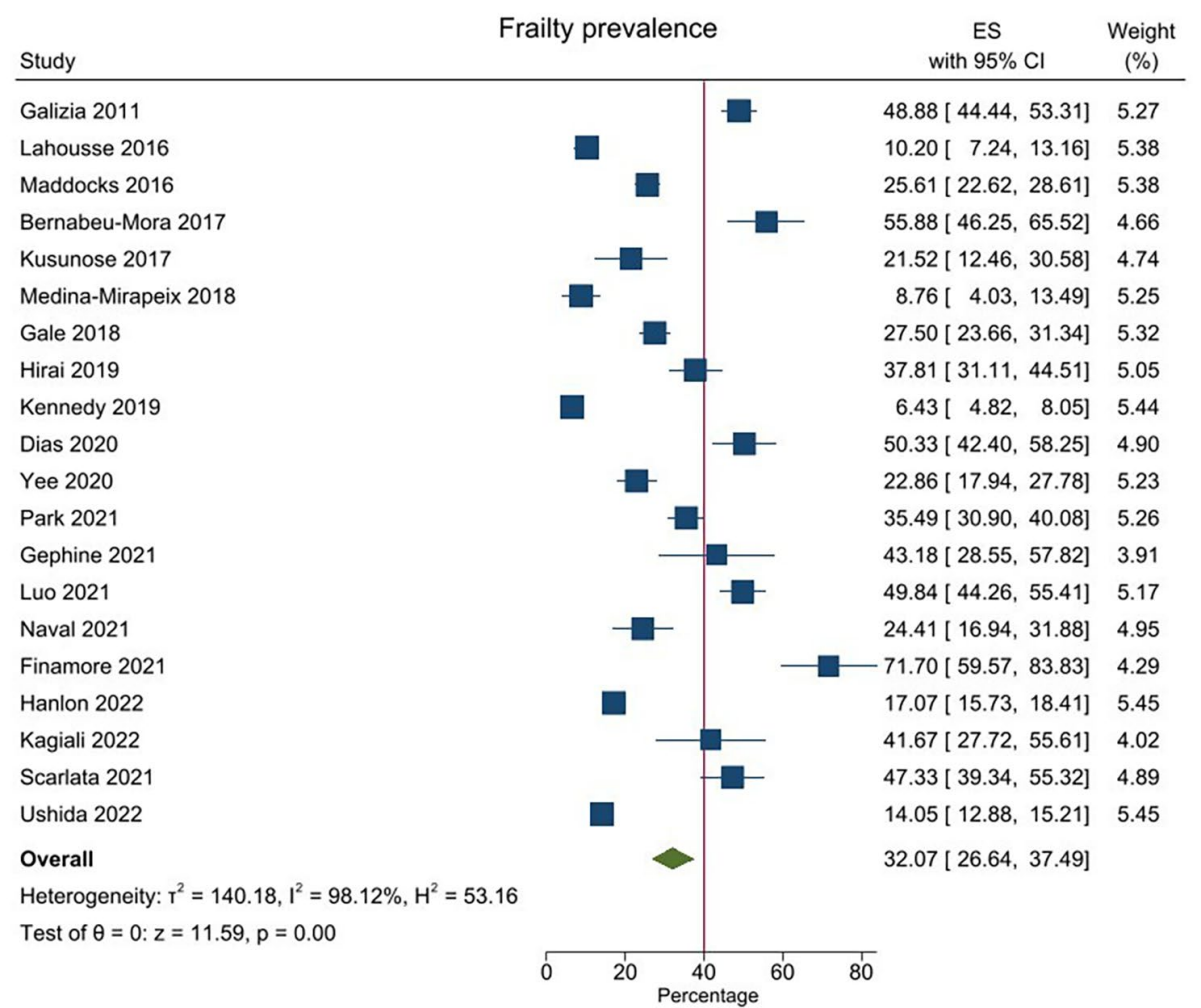
Prevalence of frailty in chronic obstructive pulmonary disease. CI, confidence interval; ES, effect size (prevalence%)

The prevalence of frailty ranged from 6.43 to 71.70% based on the frailty tool used. Overall frailty prevalence was 32.07% (95% CI 26.64–37.49; Fig. 2). The high statistical heterogeneity in this analysis (*I*^2^ = 98.12%) meant that individual study weighting was uniform (range 3.91–5.45%). Visual examination of asymmetrical funnel plots suggested publication bias (Additional Fig. 1), and Egger’s test indicated strong evidence of publication bias detected in the meta-analysis of prevalence of frailty(Z = 5.57,*P* < 0.01). Trim-and-fill analysis was performed to show the effect of the publication bias. The pooled estimate value was 26.60% (95% CI, 20.37–34.74; *P* < 0.001; random-effects model), which did not alter the significance of the results. The funnel plot after trimming is provided in Additional Fig. 7.

### Impact of frailty on clinical outcomes

**Fig. 3:**
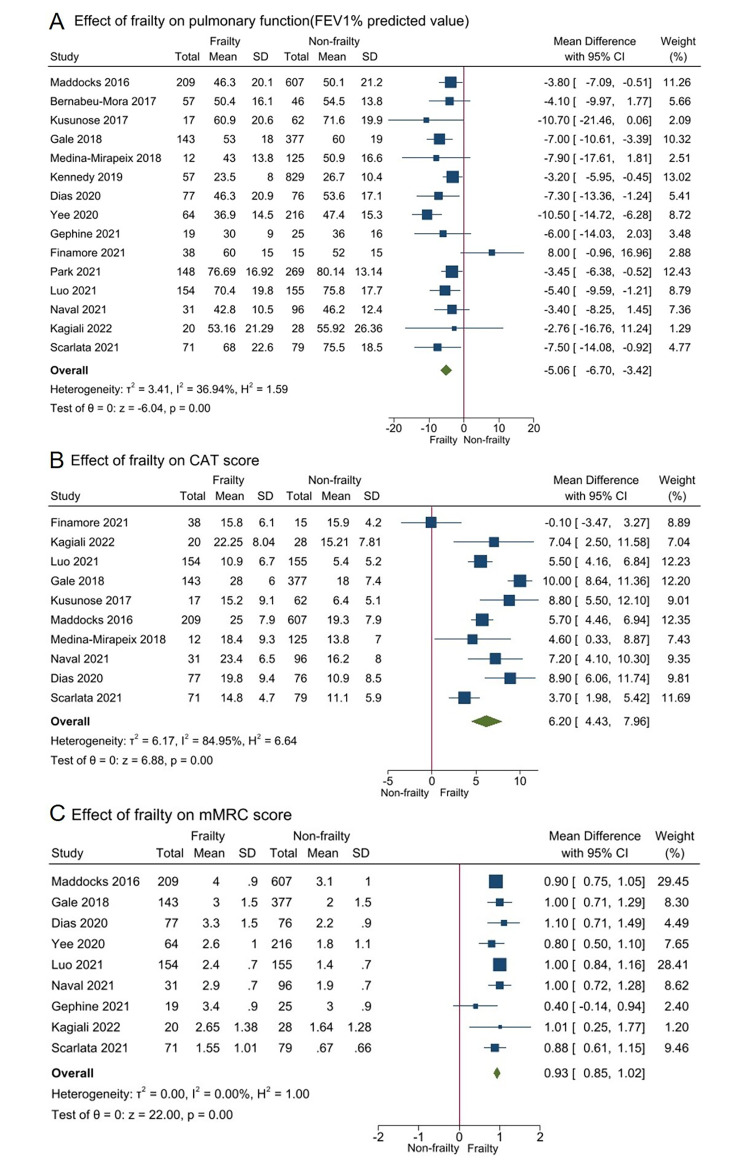
Impact of frailty on pulmonary function and dyspnea severity in individuals with COPD. COPD, chronic obstructive pulmonary disease; CAT, COPD Assessment Test; mMRC, modified Medical Research Council

Data from 15 studies [10, 11, 13, 14, 22–27, 29, 32, 33, 35, 36] involving 4,122 participants meta-analyzed showed that those with frailty presented poorer FEV1% predicted than those without frailty [mean difference − 5.06% (95%CI -6.70 to -3.42%); *I*^2^ = 36.94%, Fig. 3A].

Data from 10 studies [11, 22–24, 26, 27, 29, 32, 33, 35] involving 2,392 participants were available for meta-analysis of CAT score, showing that those with frailty presented higher CAT score than those without frailty [mean difference 6.20(95%CI 4.43 to 7.96); *I*^2^ = 84.95%, Fig. 3B]. Similarly, the meta-analysis of mMRC grade from nine studies [11, 13, 23–26, 29, 33, 35] showed that having frailty was associated with higher mMRC grade [mean difference 0.93(95%CI 0.85 to 1.02; *I*^2^ = 0.00%, Fig. 3C].

**Fig. 4:**
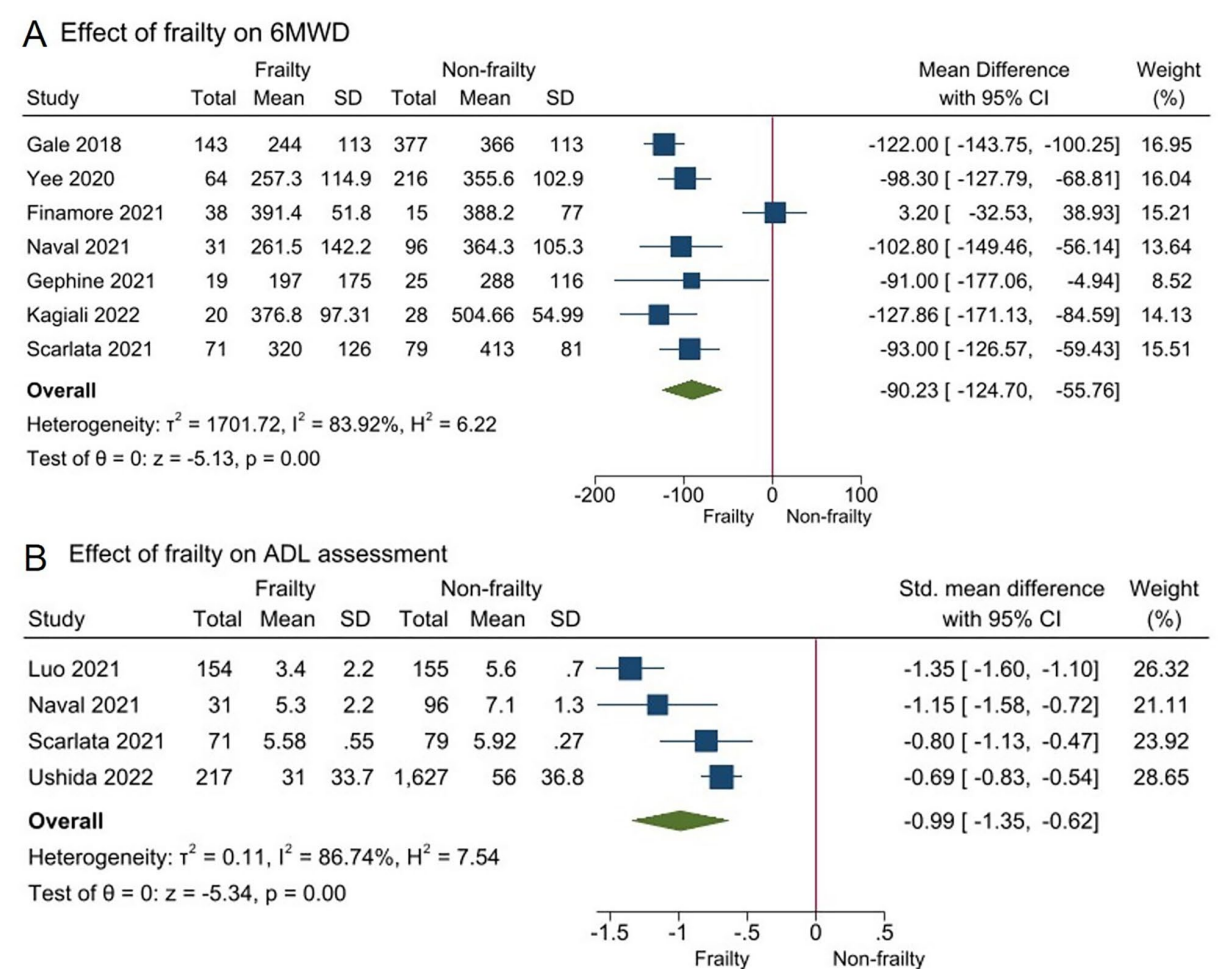
Impact of frailty on 6MWD and ADL in individuals with COPD. COPD, chronic obstructive pulmonary disease;6MWD, six minutes walking distance; ADL, activities of daily living

Seven studies evaluated the association between frailty status and 6-minute waking test [13, 22–26, 33]. Frailty was associated with shorter 6MWD [mean difference − 90.23 m (95%CI -124.70 to -55.76); *I*^2^ = 83.92%, Fig. 4A]. Four studies involving 2,430 participants reported data on activities of daily living via the Katz Activities of Daily Living [11, 33], Lawton scale [11, 24, 33], Barthel index [12]. Having frailty was associated with poorer ADL [SMD − 0.99 (95%CI -1.35 to -0.62); *I*^2^ = 86.74%, Fig. 4B].

**Fig. 5:**
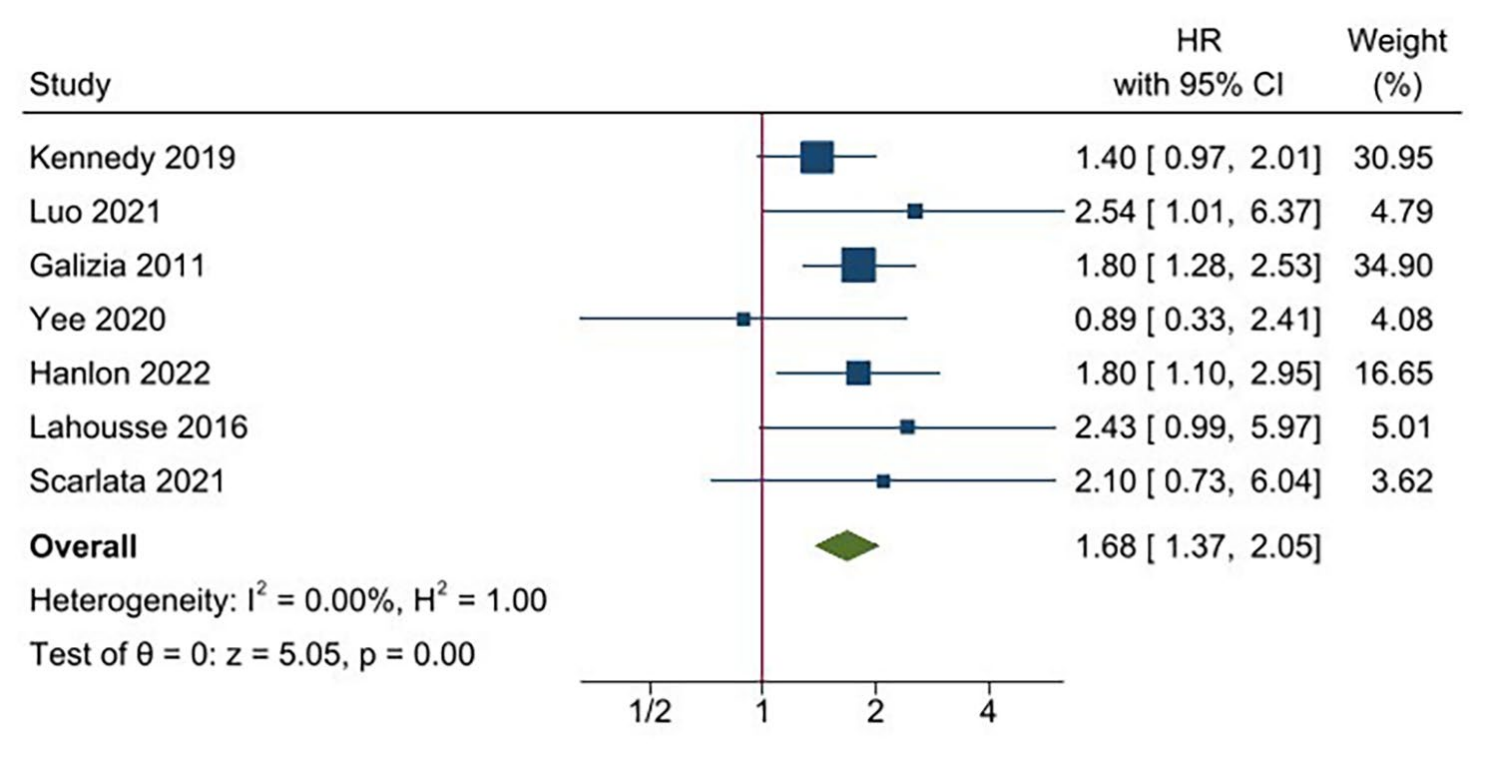
Impact of frailty on mortality in individuals with COPD. COPD, chronic obstructive pulmonary disease

The overall pooled analysis of the 7 studies [10, 11, 13, 30, 31, 33, 34]demonstrated a 1.68-fold increase in the risk of long-term all-cause mortality for frail patients (95% CI 1.37–2.05; *P* < 0.0001) compared with non-frail patients (Fig. 5). No significant heterogeneity among the 7 studies was observed (*P* = 0.60, *I*^2^ = 0.00%). The results of the funnel plot suggested little publication bias for the above analyses of frailty upon clinical outcomes (Additional Figs. 2–6).

A summary of findings related to the rehospitalization and acute exacerbation is presented in Table 2; Quantitative meta-analysis was not possible due to lack of sufficient data. Compared with non-frail individuals, those with frailty tend to have heightened risk of rehospitalization [10, 11, 30]. Only two studies examined acute exacerbation risks for frailty COPD patients. Of them, Halon et al. [30] found that frailty increased the risk of hospitalized exacerbation and community exacerbation adjusting for FEV1. On the contrary, the other study showed that the frailty measured by FFP was not associated with COPD exacerbations [13].

**Table 2:** Clinical impact of the frailty in rehospitalization and acute exacerbation

	Frailty measurement	Compared with individuals with COPD without frailty
		frailty
Rehospitalization	FFP	Higher risk [10, 11, 30]/ N.d [13]
	HFRS	N.d [12]
Acute exacerbation	FFP	Higher risk [30] /N.d [13]

**COPD** Chronic obstructive pulmonary disease, **FFP** Fried frailty phenotype, **HFRS** Hospital Frailty Risk Score, **N.d** not significant difference

## Discussion

This meta-analysis evaluated the impact of frailty on health outcomes related to pulmonary function, symptom burden, physical function, and risk of mortality in patients with COPD. The quality of included most studies was grouped in terms of moderate to low risk of bias. The main finding of our meta-analysis is that frailty is associated with reduced FEV1% predicted, higher CAT score and mMRC grade, shorter six minutes walking distance (6MWD) and poorer ADL; patients with COPD and frailty had a higher risk of long-term all-cause mortality. Therefore, frailty is a prospective predictor in the risk classification of COPD.

In this review, the proportion of patients with COPD and frailty ranged from 6.43 to 71.70%. However, frailty prevalence in most studies ranged from 20 to 50%. Frailty has been estimated as occurring in up to 19% of people with stable COPD [9] in previous study and more than 50% of patients with acute exacerbations of COPD (AECOPD) [14]. The difference in the prevalence of frailty may be due to the heterogeneity of frailty assessment tools and the severity of COPD.

The pathophysiological mechanism of frailty is multidimensional including higher chronic inflammation, immune activation, dysregulation of the musculoskeletal and endocrine systems and higher level of oxidative stress [37]. A growing evidence supports the contribution of chronic inflammation and immune system dysfunction to frailty [38, 39]. Inflammation may accelerate the catabolism of skeletal muscle and adipose tissue, inducing the muscle weakness and weight loss that are symbols of frailty [40]. Patients with COPD also show signs of chronic inflammation; higher levels of systemic proinflammation biomarkers are associated with poorer outcomes [41]. Frailty patients often have decline in the ability to cough and weak cough diminishes the ability of airway clearance. In COPD patients, weak cough is associated with increased two-year mortality after a scheduled extubation [42].

Although there is no consensus on which frailty measures are most suitable for patients with COPD at present. FFP is still a widely accepted reference model [43]. This was further confirmed in 50% of the included studies where the FFP was used as the measurement [10, 11, 13, 23–25, 29–32]. However, the participants in the above studies focused on the community-based population and stable outpatients with COPD. For patients with advanced and critical lung disease, FFP has proven limited utility [44]. HFRS [12] and REFS [14] were used to predict the outcome of patients with AECOPD in previous studies. Frailty measured by REFS can predict the risk of early hospital readmission in patients hospitalized for AECOPD [14]. HFRS was associated with prolonged hospitalization, but had poor predictive performance of mortality after adjusting for covariates [12]. It is reasonable to consider using various tools for different health status of COPD to evaluate the effect of frailty on outcomes in clinical practice.

Frailty is an increasingly recognized and potential therapeutic risk factor in acute exacerbations of chronic airway diseases [45]. If physical frailty is present, comprehensive and multicomponent interventions except for respiratory drug therapy seem necessary. Rehabilitation serves as an important component of the management of COPD. Pulmonary rehabilitation (PR) can significantly improve a range of clinical outcomes in frail patients with COPD, including symptom burden (mMRC grade and CAT score), exercise performance, physical activity level and health status in the short term [29]. For frail COPD patients with chronic respiratory failure, these benefits were maintained more than 6 months after the end of PR [46]. Physical frailty was not a barrier for benefiting from the intervention. Indeed, physical frailty can be reversed from PR intervention at least partially. After rehabilitation, more than half of previously frail patients improved their frailty status [46]. Future research studies are needed to determine the most effective PR program and the effect in frail patients with COPD in the long term.

The results of our meta-analysis highlight that frailty evaluation may improve risk stratification in patients with COPD. Comprehensive geriatric assessment is proven beneficial to the management of frail patients, which increases possibility of being alive and returning homes after an emergency admission to hospital [47]. Frailty is common in patients with COPD and associated with poorer clinical outcomes. Clinicians should stratify patients according to their frailty status and take timely interventions, which may reverse the frailty status and improve the prognosis of patients with COPD especially in the older adults. Notably, clinicians should be aware of the importance of PR for frail patients with COPD.

There are several limitations that should be noted. First, although this review had included studies to investigate the prevalence of frailty in COPD, the funnel plot suggested the publication bias. After correction of the publication bias by the trim-and-fill method, the pooled estimate value (26.60%) was slightly lower than the original 32.07%, whereas the difference remained statistically significant. Secondly, different studies used different measures of frailty. The inadequate and inconsistent definition of frail status may affect the predictive value of frailty. Thirdly, the follow-up time ranged from 90 days to 12 years, with most of studies focused on the long-term mortality. Therefore, no further analysis was made to investigate the impact of frailty on short-term mortality. Future studies are warranted to investigate the correlation between frailty and AECOPD. Finally, this review was unable to elucidate the direct relationship between frailty and readmission and acute exacerbation due to a lack of data.

### Conclusion

Frailty is prevalent in people with COPD and negatively impacts clinical outcomes. Assessment of the frailty status of patients with COPD can potentially guide clinical management of this population. Patients living with COPD and frailty may benefit from some interventions such as pulmonary rehabilitation.

## Electronic supplementary material

Below is the link to the electronic supplementary material.

Supplementary Material 1

## Data Availability

All data generated or analyzed during this study are included in this published article. The data could be freely available for anyone interested.

## Abbreviations

ADL: activities of daily living
AECOPD: acute exacerbation of chronic obstructive pulmonary disease
AHRQ: Agency for Healthcare Research and Quality
COPD: chronic obstructive pulmonary disease
CI: confidence interval
CAT: COPD Assessment Test
CFS: clinical frailty scale
CGA: the comprehensive geriatric assessment
FFP: the Fried frailty phenotype
mMRC: modified Medical Research Council
NOS: Newcastle-Ottawa Quality Assessment Scale
HR: hazard ratio
HFRS: the Hospital Frailty Risk Score
PR: pulmonary rehabilitation
REFS: Reported Edmonton Frail Scale
SMD: standardized mean difference
TUG: Timed “Up and Go” test
6MWD: six minutes walking distance
