# Supplementary material for: Prevalence and clinical impact of frailty in COPD: a systematic review and meta-analysis
Source: BMC Pulm Med. 2023 May 12;23:164. doi: 10.1186/s12890-023-02454-z (PMC10182679; doi:10.1186/s12890-023-02454-z)
Supplement: Supplementary file 1 — Supplementary Material 1 [file 12890_2023_2454_MOESM1_ESM.docx]

# SUPPLEMENTARY CONTENTS

**Additional Table 1: Search strategy**

**Additional Table 2. Quality assessment of the cohort studies using the Newcastle-Ottawa Scale**

**Additional Table 3. Quality assessment of the cross-sectional studies using the Agency for Healthcare Research and Quality tool**

**Additional Table 4. Quality assessment of the randomized controlled trial using the Cochrane Collaboration’s tool.**

**Additional Figure 1. Funnel plot of meta-analysis for prevalence of frailty.**

**Additional Figure** **2. Funnel plot of meta-analysis for pulmonary function.**

**Additional Figure 3. Funnel plot of meta-analysis for CAT score.**

**Additional Figure** **4. Funnel plot of meta-analysis for mMRC score.**

**Additional Figure 5. Funnel plot of meta-analysis for 6MWD.**

**Additional Figure 6. Funnel plot of meta-analysis for mortality.**

**Additional Figure 7. Funnel plot after use of the trim-and-fill method.**

**Additional Table 1. Search strategy**

| Database | Search number | Search Details |
| --- | --- | --- |
| Pubmed | #1 | "pulmonary disease, chronic obstructive"[MeSH Terms] |
|  | #2 | "chronic obstructive bronchitis"[Text Word] |
|  | #3 | "chronic obstructive pulmonary disease"[Text Word] |
|  | #4 | "COPD"[Text Word] |
|  | #5 | "chronic obstructive airway disease"[Text Word] |
|  | #6 | "chronic obstructive lung disease"[Text Word] |
|  | #7 | "emphysema"[Text Word] |
|  | #8 | "bronchitis"[Text Word] |
|  | #9 | "pulmonary disease, chronic obstructive"[MeSH Terms] OR "chronic obstructive bronchitis"[Text Word] OR "chronic obstructive pulmonary disease"[Text Word] OR "COPD"[Text Word] OR "chronic obstructive airway disease"[Text Word] OR "chronic obstructive lung disease"[Text Word] OR "emphysema"[Text Word] OR "Bronchitis"[Text Word] |
|  | #10 | "frailty"[MeSH Terms] |
|  | #11 | "Frailty"[MeSH Terms] |
|  | #12 | "frail elderly"[Text Word] |
|  | #13 | "frail"[Text Word] |
|  | #14 | "Frailty"[MeSH Terms] OR "frail elderly"[Text Word] OR "frail"[Text Word] |
|  | #15 | ("pulmonary disease, chronic obstructive"[MeSH Terms] OR "chronic obstructive bronchitis"[Text Word] OR "chronic obstructive pulmonary disease"[Text Word] OR "COPD"[Text Word] OR "chronic obstructive airway disease"[Text Word] OR "chronic obstructive lung disease"[Text Word] OR "emphysema"[Text Word] OR "Bronchitis"[Text Word]) AND ("Frailty"[MeSH Terms] OR "frail elderly"[Text Word] OR "frail"[Text Word]) AND 2002/01/01:2022/07/01[Date - Publication] |
| EMBASE | #1 | 'chronic obstructive lung disease'/exp |
|  | #2 | 'chronic obstructive pulmonary disease':kw |
|  | #3 | 'copd':kw |
|  | #4 | 'chronic obstructive airway disease':kw |
|  | #5 | 'chronic obstructive bronchitis':kw |
|  | #6 | 'pulmonary disease':kw |
|  | #7 | 'emphysema':kw |
|  | #8 | 'bronchitis':kw |
|  | #9 | #1 OR #2 OR #3 OR #4 OR #5 OR #6 OR #7 OR #8 |
|  | #10 | 'frailty'/exp |
|  | #11 | 'frail elderly':kw |
|  | #12 | 'frail':kw |
|  | #13 | #10 OR #11 OR #12 |
|  | #14 | #9 AND #13 |
|  | #15 | #9 AND #13 AND [01-01-2002]/sd NOT [02-07-2022]/sd |
| The Cochrane Library | #1 | [Pulmonary Disease, Chronic Obstructive] explode all trees |
|  | #2 | (Chronic Obstructive Bronchitis):kw |
|  | #3 | (chronic obstructive pulmonary disease):kw |
|  | #4 | (COPD):kw |
|  | #5 | (Chronic Obstructive Airway Disease):kw |
|  | #6 | (Chronic Obstructive Lung Disease):kw |
|  | #7 | emphysema:kw |
|  | #8 | (bronchitis):kw |
|  | #9 | #1 or #2 or #3 or #4 or #5 or #6 or #7 or #8 |
|  | #10 | [Frailty] explode all trees |
|  | #11 | frail:kw |
|  | #12 | (frail elderly):kw |
|  | #13 | #10 or #11 or #12 |
|  | #14 | #9 and #13 with Cochrane Library publication date Between Jan 2002 and Jul 2022 |
| Web of science | #1 | ((TS=(frailty)) OR AK=(frail)) OR AK=(frailty elderly) |
|  | #2 | (((((((TS=(Pulmonary Disease, Chronic Obstructive)) OR AK=(Chronic Obstructive Bronchitis)) OR AK=(chronic obstructive pulmonary disease)) OR AK=(COPD)) OR AK=(Chronic Obstructive Airway Disease)) OR AK=(Chronic Obstructive Lung Disease)) OR AK=(emphysema)) OR AK=(bronchitis) |
|  | #3 | #2 AND #1 |

**Additional Table 2. Quality assessment of the cohort studies using the Newcastle-Ottawa Scale**

| Study | **Questions of the Quality Assessment Tool for Cohort Studies** | | | | | | | | Score (0-9) |
| --- | --- | --- | --- | --- | --- | --- | --- | --- | --- |
|  | Representativeness of the Exposed Cohort | Selection of the Nonexposed Cohort | Ascertainment of Exposure | Outcome of Interest Not Present at Start of Study | Comparability | Assessment of Outcome | Adequacy of Duration of Follow-up | Adequacy of Completeness of Follow-up |  |
| Kennedy2019 | 1 | 1 | 1 | 1 | 2 | 1 | 1 | 1 | 9 |
| Luo2021 | 1 | 1 | 1 | 1 | 2 | 1 | 1 | 1 | 9 |
| Maddocks2016 | 1 | 1 | 1 | 1 | 1 | 1 | 1 | 1 | 8 |
| Yee2020 | 1 | 1 | 1 | 1 | 1 | 1 | 1 | 1 | 8 |
| Bernabeu-Mora2017 | 1 | 1 | 1 | 1 | 2 | 1 | 1 | 1 | 9 |
| Hanlon2022 | 0 | 1 | 1 | 1 | 2 | 1 | 1 | 1 | 8 |
| Lahousse2016 | 1 | 1 | 1 | 1 | 2 | 1 | 1 | 1 | 9 |
| Scarlata2021 | 0 | 1 | 1 | 1 | 1 | 1 | 1 | 1 | 7 |
| Ushida2022 | 0 | 1 | 1 | 1 | 2 | 1 | 1 | 1 | 8 |
| Galizia2011 | 1 | 1 | 1 | 1 | 2 | 1 | 1 | 1 | 9 |

**Additional Table 3. Quality assessment of the cross-sectional studies using the Agency for Healthcare Research and Quality tool**

| Study | **Questions of the Quality Assessment Tool for Cross-Sectional Studies** | | | | | | | | | | | Total score  (0-11) |
| --- | --- | --- | --- | --- | --- | --- | --- | --- | --- | --- | --- | --- |
|  | 1 | 2 | 3 | 4 | 5 | 6 | 7 | 8 | 9 | 10 | 11 |  |
| Kagiali 2022 | Yes | Yes | Yes | Yes | No | Yes | No | Unclear | Unclear | No | Unclear | 6 |
| Kusunose2017 | Yes | Yes | Yes | Yes | No | Yes | No | Yes | Unclear | No | Unclear | 7 |
| Naval2021 | Yes | Yes | Yes | Yes | No | Yes | No | Yes | Unclear | No | Unclear | 7 |
| Dias2020 | Yes | Yes | Yes | Yes | No | Yes | Yes | Yes | Unclear | No | Unclear | 8 |
| Gale2018 | Yes | Yes | No | Yes | No | Yes | No | Yes | Unclear | No | Unclear | 6 |
| Hirai2019 | Yes | Yes | Yes | Yes | No | Yes | No | Unclear | Unclear | No | Unclear | 6 |
| Medina-Mirapeix2018 | Yes | Yes | Yes | Yes | No | Yes | Yes | Yes | Unclear | No | Unclear | 8 |
| Park2021 | Yes | Yes | Yes | Yes | No | Yes | Yes | Yes | Unclear | No | Unclear | 8 |
| Gephine2021 | Yes | Yes | Yes | Unclear | No | Yes | No | Unclear | Unclear | No | Unclear | 5 |

**Additional Table 4. Quality assessment of the** **randomized controlled trial using the Cochrane Collaboration’s tool.**

| Study | **Quality Assessment Tool for Randomized Controlled Trials** | | | | | | Category |
| --- | --- | --- | --- | --- | --- | --- | --- |
|  | Random sequence generation | Allocation concealment | Blinding of participants and personnel | Blinding of outcome assessment | Incomplete outcome data | Selective reporting |  |
| Finamore2021 | Unclear risk | Unclear risk | High risk | Low risk | Low risk | Unclear risk | High risk |


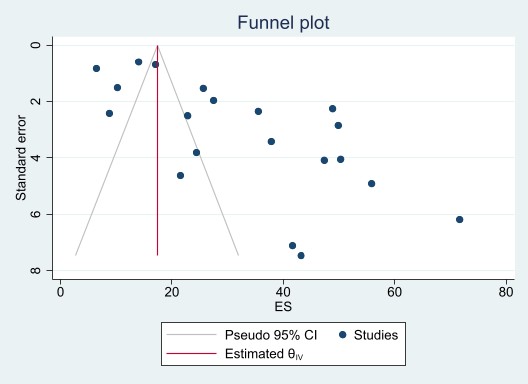


**Additional Figure 1. Funnel plot of meta-analysis for prevalence of frailty.**


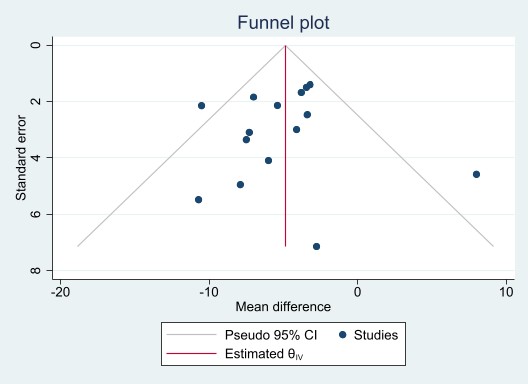


**Additional Figure** **2. Funnel plot of meta-analysis for pulmonary function.**


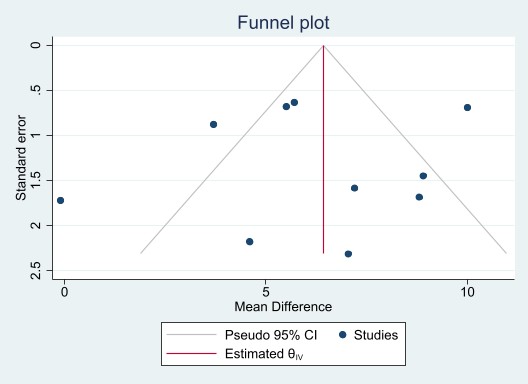


**Additional Figure 3. Funnel plot of meta-analysis for CAT score.**


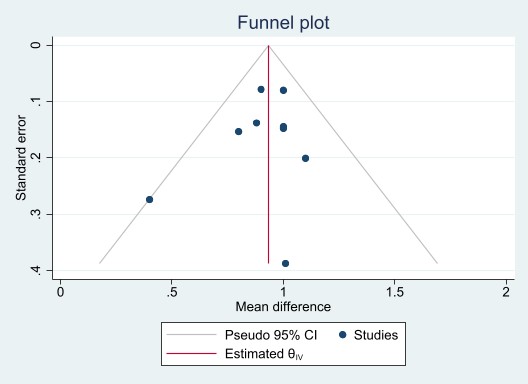


**Additional Figure** **4. Funnel plot of meta-analysis for mMRC score.**


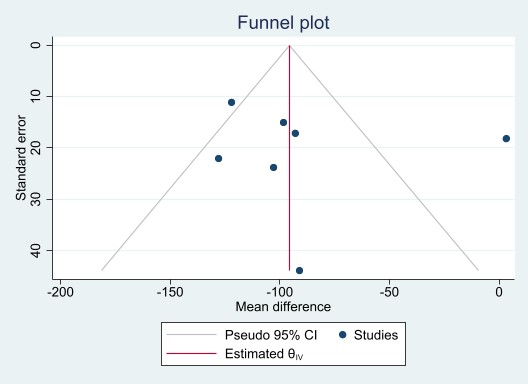


**Additional Figure 5. Funnel plot of meta-analysis for 6MWD.**


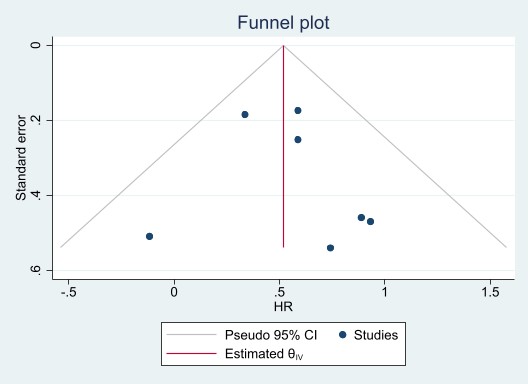


**Additional Figure** **6. Funnel plot of meta-analysis for mortality.**

**
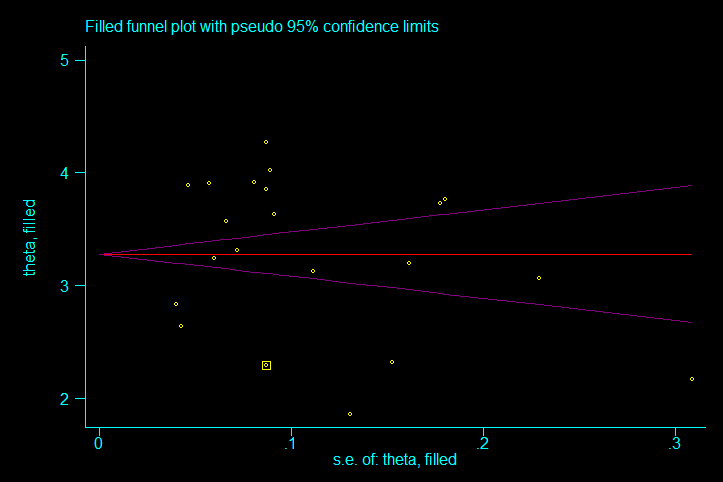
**

**Additional Figure 7. Funnel plot after use of the trim-and-fill method.**
